# Visualizing androgen signaling and assessing its interaction with canonical Wnt signaling pathways in prostate development, morphogenesis, and regeneration

**DOI:** 10.1371/journal.pgen.1011756

**Published:** 2025-06-13

**Authors:** Yao Mawulikplimi Adzavon, Dong-Hoon Lee, Alex Hiroto, Tae Ju Park, Gaeul Chu, Yunjeong Kim, Kristoffer Nikias, Cheong-Wun Kim, Dexter Hoi Long Leung, Chenmiao Liu, Hong Zeng, Zijie Sun

**Affiliations:** 1 Department of Cell Biology, Department of Oncology, and Montefiore Einstein Cancer Center, Albert Einstein College of Medicine, Bronx, New York, United States of America; 2 Department of Cancer Biology and Molecular Medicine, Cancer Center and Beckman Research Institute, City of Hope Medical Center, Duarte, California, United States of America; 3 Transgenic, Knockout and Tumor Model Center, Stanford University School of Medicine, Stanford, California, United States of America; HudsonAlpha Institute for Biotechnology, UNITED STATES OF AMERICA

## Abstract

The androgen receptor (AR) is a nuclear hormone receptor, and its activation through binding to androgens is essential for prostate development, morphogenesis, growth, and tumorigenesis. Although significant efforts have been devoted to understanding the critical role of AR, the cellular properties and functions of the AR-expressing cells acting as prostatic progenitors in controlling prostatic cell differentiation and growth still remain elusive. Additionally, dynamic paracrine interactions between urogenital mesenchyme and epithelia initiated by the AR activation through prostate development are also largely unknown. Recently, we modified the mouse *Ar* gene locus, which enables us to genetically label AR-expressing cells spatiotemporally and trace them through prostate development, morphogenesis, and growth in combination with a double-fluorescent reporter mouse model. The membrane-bound green fluorescent protein (mGFP)-expressing cells were revealed in both urogenital sinus mesenchyme (UGM) and epithelium (UGE) at embryonic day E18.5 when Tamoxifen was administrated at E13.5 to activate CreER recombinase directed by the endogenous *Ar* promoter. The AR-expressing cells and their descendants were further detected at postnatal days 10, 35, and 56, and through three cycles of prostatic regeneration by repeated androgen deprivation and replacement. Deletion of β-catenin through the AR-driven CreER in embryonic AR-expressing cells impairs prostate development and morphogenesis. Specifically, altered β-catenin expression results in loss of prostatic glandular cell polarity and activation of Fas death signaling pathways. These lines of experimental evidence demonstrate the biological relevance and significance of this new genetic tool to assess and visualize AR-mediated signaling pathways through prostatic development, growth, and tumorigenesis.

## Author summary

Prostate formation, growth, regeneration, and tumorigenesis depend on androgens and androgen receptor (AR)-mediated signaling pathways. Early tissue recombination assays demonstrated a decisive role for stromal androgen signaling in prostatic epithelium development. However, the identity of the mesenchymal cells in the urogenital sinus mesenchyme that convey androgen signaling and control prostate epithelium development, morphogenesis, and regeneration remains elusive. Additionally, how epithelial AR responds to stromal signaling to regulate differentiation, morphogenesis, and growth during embryonic development and prepubescence is unclear. Moreover, the molecular mechanisms underlying androgen signaling that initiate the paracrine regulation between prostatic mesenchymal and epithelial cells are largely unknown. One of the key bottlenecks in addressing these critical questions has been the lack of relevant Genetically Engineered Mouse Models (GEMMs) to evaluate androgen and AR action during these biological events.

To directly address this challenge, we developed a new GEMM, the *Ar* ^*IRES-CreER/Y*^ model. The fundamental difference between this model and previous ones is that AR-expressing cells can be labeled in a temporal and spatial manner. In this study, we tested AR-driven CreER activity using both a double reporter mouse strain and floxed β-catenin alleles. Deletion of β-catenin in embryonic AR-expressing cells impairs prostate development and morphogenesis. Specifically, altered β-catenin expression results in the loss of prostatic glandular cell polarity and activation of Fas death signaling pathways. These lines of experimental evidence demonstrate the relevance and significance of this new genetic tool, which will enable us to design and perform a series of experiments to fully evaluate the role and regulatory mechanisms of AR activation in prostate development, morphogenesis, growth, and tumorigenesis.

## Introduction

Androgen signaling pathways are mediated through the androgen receptor (AR) and its ligands, testosterone and 5α-dihydro-testosterone, and play an essential role in prostate development, morphogenesis, growth, and regeneration [1]. As observed in the testicular feminized (Tfm) mice, mutation of the *Ar* gene results in the complete abolition of prostate development [2]. Mutations in the human *AR* gene also result in different forms of androgen insensitivity syndrome [3]. During mouse embryogenesis, the AR is initially expressed in the urogenital sinus mesenchyme (UGM) prior to the initiation of prostate budding and morphogenesis, and, subsequently, its expression extends to the urogenital sinus epithelium (UGE) after the initiation of prostatic budding and branching morphogenesis [4–8]. Early tissue recombination experiments have elucidated a critical role of mesenchymal AR signaling in regulating prostatic epithelial development and gland formation through the paracrine interactions between the UGM and UGE compartments [9,10]. In the postnatal prostate, androgen signaling remains crucial in controlling prostatic branch morphogenesis and maturation [11]. Beyond puberty, androgen signaling continues to play an important role in regulating prostatic cell differentiation and expansion, as evidenced by prostatic regeneration through repeated cycles of androgen deprivation and replacement [6,9,12]. Recently, a significant niche role of stromal AR in Sonic Hedgehog (Shh) responsive Gli1-lineage cells has been identified in controlling mouse prostate development, morphogenesis, and growth [13,14]. However, the cellular properties and functions of the AR-expressing cells in controlling prostatic cell differentiation and growth still remain elusive. Additionally, dynamic interactions between androgens, Shh, Wnt, and other signaling pathways within prostatic mesenchyme and epithelia during the course of prostate development and growth are also largely unknown.

The AR is a member of the nuclear hormone receptor superfamily, and a ligand-regulated transcriptional factor [15]. Androgen-induced AR transcriptional activity in AR-expressing cells and their descendants controls early prostate development, morphogenesis, and growth in mice [16]. We recently developed the *Ar* ^*IRES-CreER*^ allele in the mouse *Ar* gene locus at the X chromosome using gene-targeting approaches. By combining with mouse strain bearing mTmG reporters [17], activating CreER recombinase on the *Ar* ^*IRES-CreER*^ allele by Tamoxifen administration can genetically mark AR-expressing cells in both spatial and temporal manners, enabling to visualize AR-expressing cells and trace the fate of their descendants throughout prostatic gland development, morphogenesis, and formation. In this study, we administered Tamoxifen at E13.5 to activate the CreER recombinase driven by the endogenous *Ar* promoter in mice. The membrane-bound green fluorescent protein, mGFP-expressing cells were identified in both UGM and UGE at embryonic day E18.5. The mGFP-expressing cells were further detected at postnatal days 10, 35, and 56 (P10, P35, and P56), as well as through three cycles of prostatic regeneration induced by repeated androgen deprivation and replacement. Both immunohistochemical (IHC) and immunofluorescent (IF) analyses further demonstrate the cellular properties of the above mGFP-expressing cells, implicating the prostatic progenitor properties of embryonic AR-expressing cells. Disruption of canonical Wnt signaling pathways by deleting β-catenin expression using the AR-driven CreER recombinase in AR-expressing cells at E13.5 significantly impairs prostatic epithelial budding, as well as glandular development and morphogenesis. Specifically, deletion of β-catenin expression results in a loss of prostatic glandular cell polarity, which further activates Fas death signaling pathways. These data provide new insight into Wnt/β-catenin signaling in AR-expressing cells to control prostate early development and pubertal morphogenesis and growth. Results from this study also demonstrate the relevance and significance of this new genetic tool for visualizing and assessing AR-mediated signaling pathways *in vivo*.

## Results

### Developing the *Ar* ^*IRES-CreER*^ allele to genetically label embryonic AR-expressing cells

**Fig 1:**
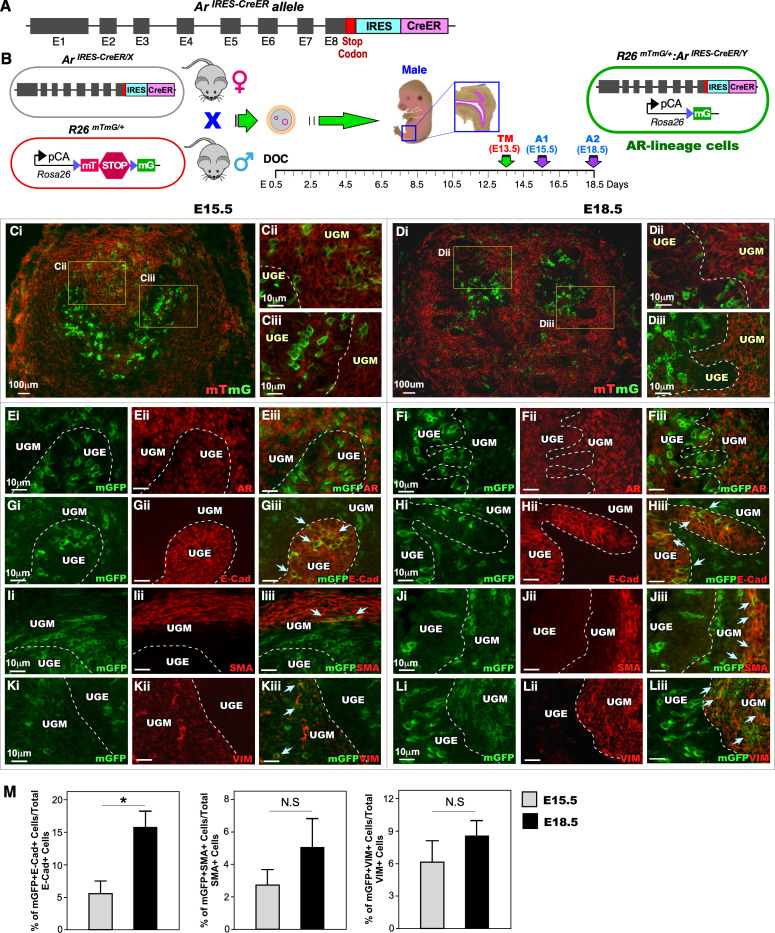
Generation and characterization of early prostate development of *Ar*^*IRES-CreER*^ mice. (A) Schematic of the targeted *Ar* allele displaying the inserted IRES and CreER sequences. (B) Diagram of the *Ar*^*IRES-CreER*^ and *R26*^*mTmG/+*^ alleles relative to the mating strategy. Following the day of conception (DOC), a timeline indicates the analysis timepoints. The construct depicts the recombination event occurring in AR-expressing cells, resulting in a switch from red to green fluorescence. (C-D) Representative mTmG assay images showing AR-lineage cells (green) at E15.5 (C) and E18.5 (D). (E-K) Representative immunofluorescence staining for GFP, AR, and lineage-specific markers of epithelial and mesenchymal cells using the indicated proteins/antibodies. (M) Graph showing the proportion of AR-lineage cells within the epithelial and mesenchymal compartments at E15.5 compared to E18.5. Each timepoint includes analysis of at least four urogenital sinus (UGS) samples. White dashed lines delineate the boundaries between the UGS epithelium and the surrounding mesenchyme. All scale bars are as indicated. *: p < 0.05; NS: non-significant.

Using gene targeting approaches, we generated a new mouse model, termed *Ar* ^*IRES-CreER*^ mice, in which the *CreER* recombinase sequence was inserted into the 3’ untranslated region of the mouse *Ar* gene locus on the X chromosome via an engineered internal ribosome entry site (IRES, Fig 1A). In this mouse model, the transcription of *CreER* recombinase is regulated by the endogenous *Ar* gene promoter. Expressed CreER recombinase can be conditionally activated by Tamoxifen, a synthetic ER ligand, to activate *Cre-loxP* recombination. Therefore, the current *Ar* ^*IRES-CreER*^ mouse model differs from the previous *Ar* ^*IRES-Cre*^ model [8], and provides both spatial and temporal controls through the endogenous *Ar* gene promoter to activate *CreER*-mediated recombination. Both male and female heterozygotes and homozygotes *Ar* ^*IRES-CreER*^ mice appeared viable and fertile, with no visible abnormalities. To test *CreER/LoxP* mediated gene recombination, female *Ar* ^*IRES-CreER/X*^ mice were mated with male *Rosa26* ^*mTmG/*+^ (*R26* ^*mTmG/*+^) reporter mice, a double-fluorescent reporter mouse model [17], to generate male *R26R*^*mTmG/*+^*:Ar* ^*IRES-CreER/Y*^ mice (Fig 1B). Since the expression of AR has been detected at embryonic day 13.5, E13.5, we administrated Tamoxifen at E13.5 to pregnant females bearing embryos of the desired genotype and analyzed male UGS tissues at E18.5 (Fig 1B). Tamoxifen-induced CreER activation resulted in spontaneous recombination of the *floxed* reporter loci, generating a permanent genetic marker by switching the expression from membrane-bound tandem dimer Tomato (mT) to mGFP [17] (Fig 1B). These genetically labeled cells not only carry the mGFP expression throughout their lifespan but also transmit it to their descendants, hereafter referred to as AR-lineage cells, enabling us to trace the fates of these cells. CreER-induced mGFP expression appears within the UGE and UGM in both E15.5 and E18.5 UGS tissues (Fig 1Ci-Diii). Co-staining of UGS tissues showed the overlays of mGFP and AR expression in cells of both UGE and UGM areas on E15.5 and E18.5 samples. Additionally, co-staining of mGFP with E-cadherin (E-Cad, Fig 1Gi-Hiii), Smooth Muscle Actin (SMA, Fig 1Ii-Jiii), or Vimentin (VIM, Fig 1Ki-Liii), appeared in either UGE or UGM areas, further confirming the epithelial and stromal cellular properties of embryonic AR-lineage cells in both E15.5 and 18.5 samples. Quantification of mGFP + E-Cad+ in total E-Cad+ cells revealed a significant increase in E18.5 samples compared to E15.5 samples (Left panel, Fig 1M). In contrast, no significant differences were observed in mGFP + SMA+ versus total SMA+ cells or mGFP + VIM+ versus total VIM+ cells between E15.5 and 18.5 samples, respectively (middle and right panels, Fig 1M). Together, these data demonstrate the cellular properties of AR-expressing cells labeled at E13.5, as well as the highly proliferative ability of AR-expressing UGE cells.

### Embryonic AR-expressing cells possess prostatic progenitor cell properties and expand during pubertal growth

**Fig 2:**
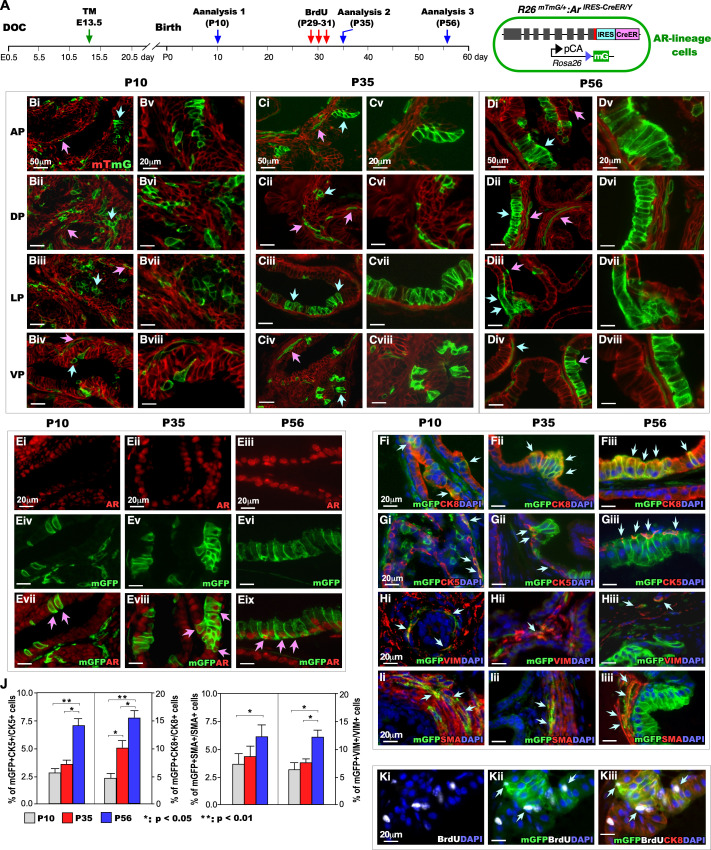
Lineage tracing of embryonic AR-expressing cells throughout postnatal prostate development. (A) Timeline illustrating the experimental procedure and analysis timepoints, along with a schematic of the genetic construct showing the Cre-mediated recombination event that permanently labels AR-lineage cells with mGFP. (Bi-Dviii) Representative images showing the clonal expansion of embryonic mGFP-labeled AR-lineage cells (green) during prostate morphogenesis (P10), prepubertal development (P35), and postpubertal growth (P56). (Ei-Eix) Representative images showing the expression of AR and mGFP in prostate tissues isolated at postnatal days P10, P35, and P56. Pink arrows indicate cells positive for both AR and mGFP. (Fi-Iiii) Characterization of the cellular properties of embryonic AR-lineage cells (mGFP+ cells) using different cellular markers: basal epithelial cell marker (CK5), luminal epithelial cell marker (CK8), fibroblast marker (VIM), and smooth muscle cell marker (SMA). Light blue arrows indicate triple-positive cells. (J) Graph showing the percentage of double-positive cells (mGFP+ with CK5, CK8, VIM, or SMA) per total CK5 + , CK8 + , VIM + , or SMA+ cells at the three different time points. *: p < 0.05; **: p < 0.01. (Ki-Kiii) Representative images of co- or triple-IF analyses showing BrdU-positive cells stained with DAPI, mGFP, and CK8 to assess proliferative cell properties.

To assess the fate and function of the embryonic AR-expressing cells labeled at E13.5, we traced these mGFP-expressing cells, also termed AR-lineage cells, at postnatal P10, prior to prostatic morphogenesis; at P35, during pubertal prostatic growth; and at P56, after puberty in male *R26*^*mTmG/*+^*:Ar*^*IRES-CreER/Y*^ mice [18–20](Fig 2A). We observed mGFP-expressing cells in four individual prostatic lobes, including anterior (AP), dorsal (DP), lateral (LP), and ventral (VP), in P10, 35, and 56 samples (Fig 2Bi-Dviii). Both epithelial and stromal mGFP-expressing cells (indicated by light blue and pink arrows, respectively) were identified in different prostatic lobes at P10 (Fig 2Bi-iv), P35 (Fig 2Ci-iv), and P56 (Fig 2Di-iv). Using co-immunofluorescent (Co-IF) approaches, we further assessed the expression of the AR in these postnatal AR-lineage cells (Fig 2Ei-ix). Cells positive for both mGFP and AR antibodies were detected in P10, P35, and P56 samples (Pink arrows, Fig 2Evii-ix). Subsequently, using triple-immunofluorescent (Triple-IF) assays, we further examined the cellular properties of the AR-lineage cells in postnatal prostate samples. Positive staining of mGFP and DAPI, with Keratin5 (CK5), Keratin8 (CK8), SMA, or VIM antibodies appeared in P10, P35, and P56 samples (Fig 2Fi-Iiii), affirming the cellular properties and differentiation fates of the descendants of embryonic AR-expressing cells. Measuring double mGFP + CK5+ or mGFP + CK8+ in total CK5+ or CK8 + cells showed a significant increase at P56 compared to P10 or P35 samples, respectively (Fig 2J). Additionally, an increase of mGFP + CK8 + cells also appeared between P10 and P35 samples (Fig 2J). Similarly, analysis of stromal AR-lineage cells demonstrated a significant increase in the proportion of mGFP + VIM+ cells among total VIM+ cells at P56 compared to P10 or P35 samples (Fig 2J). In contrast, a significant increase of mGFP + SMA+ cells relative to total SMA+ cells was only observed between P10 and P56 samples (Fig 2J). These observations implicate the proliferative ability of AR-lineage cells in response to rising androgen levels during puberty in both prostatic epithelial and stromal compartments. Notably, prostatic luminal epithelial cells showed high cellular growth and expansion throughout puberty, further underscoring their androgen-dependent growth properties. To directly assess the cell proliferative properties of androgen-lineage cells, we injected BrdU three constitutive times between P29 and P31 and analyzed the prostate samples at P56. BrdU-positive staining was detected in DAPI+ and mGFP+ cells (light blue arrows, Fig 2Ki-ii), some of which also showed positive staining for CK8+ (light blue arrows, Fig 2Kiii), further demonstrating the high proliferative properties of prostatic luminal epithelial cells.

### Embryonically labeled AR-expressing cells possess regenerative ability through repeated cycles of androgen deprivation and replacement

**Fig 3:**
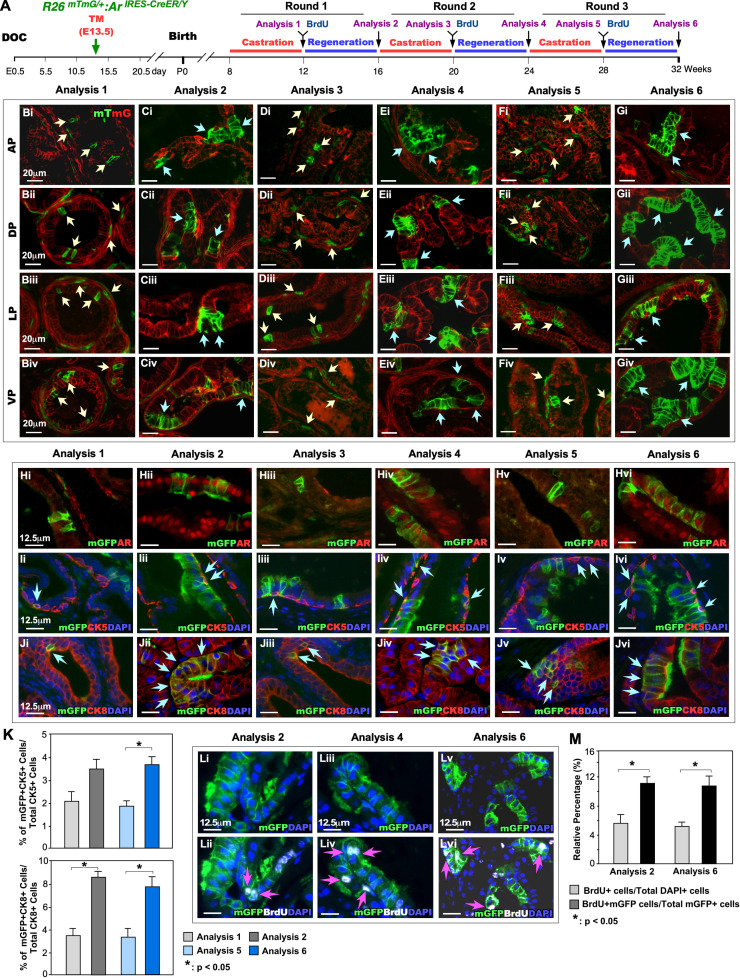
Regenerative potential of AR-lineage cells. (A) Timeline illustrating the experimental procedure, including multiple rounds of regression-regeneration assays, with corresponding analysis timepoints. (Bi-Giv) Representative images showing the clonal expansion of embryonic mGFP-labeled AR-lineage cells in four different prostate lobes, including AP: anterior prostate; DP: dorsal prostate; LP: lateral prostate; VP: ventral prostate, following multiple cycles of androgen depletion and restoration. Yellow arrows indicate mGFP-positive cells in samples isolated at the end of castration, whereas light blue arrows indicate mGFP-positive cells in samples isolated at the end of regeneration. (Hi-Hvi) Representative images showing AR expression in these samples. (I1i-Ivi and K) Images demonstrating cellular identity, and (Li-Lvi & M) Images and quantification showing the proliferative capacity of embryonic AR-lineage cells in response to androgen depletion and restoration. *: p < 0.05; **: p < 0.01.

Emerging evidence has shown that prostatic stem/progenitor cells in adult mice can survive androgen withdrawal and enable to regenerate prostatic glands when androgens are supplemented [6,9]. However, the cellular identity of these prostatic stem/progenitor cells remains unclear. Specifically, the status of AR expression in prostatic stem and progenitor cells also remains elusive. To address these important questions, we assessed the regenerative ability of embryonic AR-expressing cells and their descendent cells throughout multiple rounds of androgen withdrawal and supplementation (Fig 3A). In these experiments, we specifically focused on the regenerative capacity of post-puberty AR-lineage cells in prostatic epithelia based on the results of the above cell tracing experiments (Fig 1). Male *R26*^*mTmG/*+^*:Ar* ^*IRES-CreER/Y*^ mice received Tamoxifen at E13.5 were born, castrated at 8 weeks, and then administrated BrdU and androgen pellets at 12 weeks (Fig 3A). This castration-regeneration cycle was repeated twice with an eight-week interval. At the end of each castration or regeneration period, the mouse prostate tissues were isolated for analyses (Fig 3A). AR-lineage cells bearing mGFP expression were detected in all prostatic lobes, including AP, DP, LP, and VP, in the samples isolated at each castration and regeneration cycle (Fig 3Bi-Giv). Notably, individual mGFP+ cells were more apparent in castrated samples (yellow arrows, Fig 3Bi-iv, 3Di-iv, and 3Fi-iv) but clusters of mGFP+ cells were revealed in all prostatic lobes of prostate tissues isolated at the end of regeneration cycles (light blue arrows, Fig 3Ci-iv, 3Ei-iv, and 3Gi-iv), evidencing the expansion of AR-lineage cells in response to androgen supplements. Analysis of AR expression showed that most prostatic epithelial mGFP+ cells express AR in both castrated and regenerative samples (Fig 3Hi-vi). Triple-IF analyses identified both mGFP + CK5 basal cells (Fig 3Ii-vi) and mGFP + CK8 + luminal epithelial cells (Fig 3Ji-vi) in all prostate tissues isolated from castrated or regenerated mice. Quantitative analyses revealed that the proportion of mGFP + CK5 + cells among total CK5 + cells was significantly increased in the third castration and regeneration cycle compared to the first (right panel, Fig 3K), whereas the proportion of mGFP + CK8 + cells among total CK8 + cells showed a significant increase in both castrated and regenerated samples between the first and third rounds (left panel, Fig 3K). These data are consistent with the previous observations in P56 samples (see Fig 2), and further demonstrate the high androgen-induced cell proliferative ability of prostatic luminal AR-lineage cells. To directly examine the cell proliferation ability through repeated cycles of androgen deprivation and replacement, we administered BrdU at the end of each castration period (Fig 3A) and performed Co-IF assays to assess BrdU expression in prostate tissues isolated from regenerated mice. BrdU + mGFP+ cells were detected in all regenerated prostate tissues, affirming the proliferative ability of AR-lineage cells in response to androgens (Fig 3Li-vi). Quantitative analysis showed a significant increase in the percentage of BrdU + mGFP+ cells among total mGFP+ cells in comparison to the one of BrdU+ cells among total DAPI+ cells in the prostate tissue samples isolated from both the first and third cycles (Fig 3M), demonstrating higher proliferative ability of AR-lineage cells than the overall cell population. These experimental results provide direct evidence indicating that AR-lineage cells possess prostatic progenitor properties and are capable of proliferating and expanding through multiple cycles of castration and regeneration in response to supplemental androgens.

### Deletion of β-catenin in embryonic AR-expressing cells impairs early prostate development

**Fig 4:**
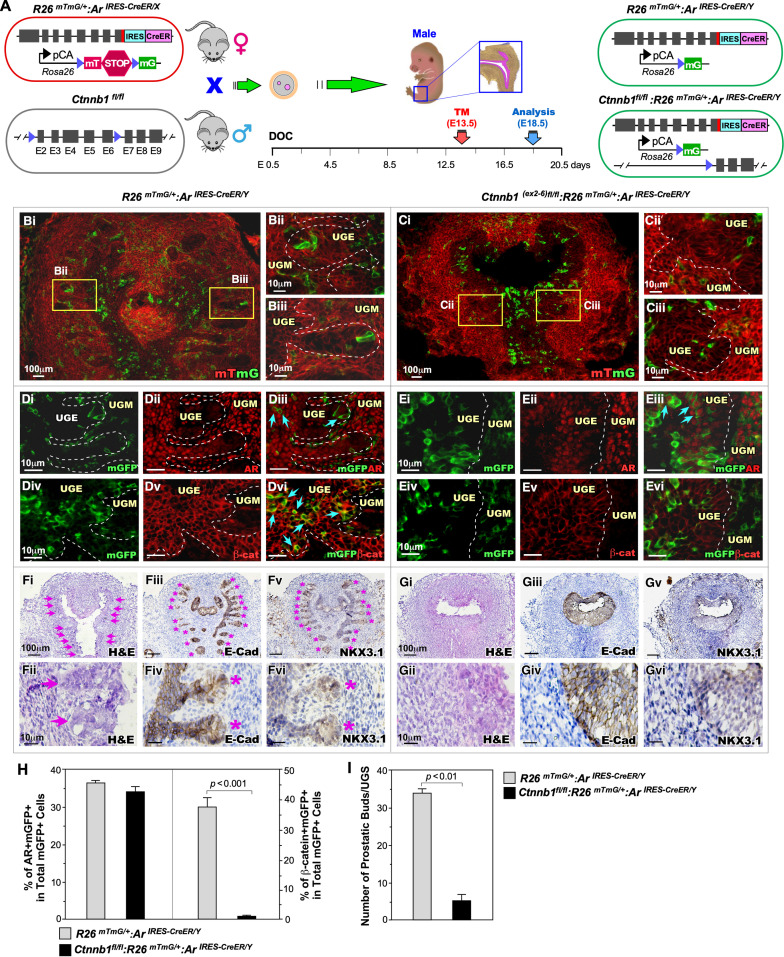
β-catenin is required in AR-lineage cells for early prostate development. (A) Schematic illustrating the genetic construct and mating strategy to efficiently knock out (KO) β-catenin in AR-lineage cells. (Bi–Ciii) Representative mTmG assay images showing AR-lineage cells (green) in the urogenital sinus (UGS) of *R26*^*mTmG/*+^*:Ar*^*CreER/Y*^ (Ctrl) and *Ctnnb1*^*fl/fl*^:*R26*^*mTmG/*+^*:Ar*^*CreER/Y*^ (β-catKO) embryos. (Di–Eiii) Co-immunofluorescence (Co-IF) for mGFP and AR confirming that mGFP⁺ cells are AR-lineage cells. (Div–Evi) Co-IF images for mGFP and β-catenin, validating effective β-catenin expression in Ctrl and deletion in β-catKO embryos. (Fi–Fii; Gi–Gii) H&E staining of UGS at E18.5. (Fiii–Fvi; Giii–Gvi) Immunostaining for the epithelial marker E-cadherin (E-Cad) and the budding marker NKX3.1 to assess epithelial identity and prostate budding. (H) Quantification of mGFP + /AR+ double-positive cells as a percentage of total mGFP+ cells in the UGS of control and β-catKO embryos. (I) Quantification of prostatic buds in control versus β-catKO UGS.

Early studies have shown a significant role of canonical Wnt signaling pathways in early prostate development, morphogenesis, and growth [21–23]. Specifically, deletion of either β-catenin or AR expression alone in prostatic Wnt-responsive Axin-expressing cells has been shown to impair early prostate development and gland formation [23,24]. To further assess the interactions between AR and β-catenin in governing prostate development and morphogenesis, we developed mice with *Ctnnb1* ^*Exon2-6fl/Exon2-6fl*^*:R26* ^*mTmG/*+^*:Ar* ^*IRES-CreER/Y*^, (*Ctnnb1*^*fl/fl*^*:R26* ^*mTmG/*+^*:Ar* ^*IRES-CreER/Y*^,β-catKO) and *R26* ^*mTmG/*+^*:Ar* ^*IRES-CreER/Y*^ (Ctrl). The pregnant females bearing embryos of the above-desired genotypes were administered Tamoxifen at embryonic day E13.5 and analyzed at E18.5 (Fig 4A). Tamoxifen-induced mGFP-expressing cells were detected in the UGE and UGM regions of UGS tissues isolated from both Ctrl and β-catKO male embryos (Fig 4Bi-iii and 4 Ci-iii). Co-IF analyses showed co-expression of mGFP and AR proteins in both β-catKO and Ctrl UGS tissues (light green arrows, Fig 4Di-iii and 4Ei-iii,). Quantification of AR + mGFP+ cells among total mGFP+ cells showed no significant difference between β-catKO and Ctrl UGS tissues (left panel, Fig 4H). However, co-expression of mGFP and β-catenin was predominantly observed in cells within the UGE region of Ctrl UGS tissues compared to their counterparts in β-catKO UGS tissues (Fig 4Div-vi, light blue arrows, and Fig 4Eiv-vi). A significant decrease in the percentage of mGFP + β-catenin+ among total mGFP+ cells appeared in UGS tissues of β-catKO compared to Ctrl mice (right panel, Fig 4H), demonstrating the specific deletion of β-catenin in embryonic AR-lineage cells.

Histological analyses of E18.5 UGS tissues from control mice showed fully developed prostatic buds with positive E-cadherin and NKX3.1 staining (pink arrows and stars, Fig 4Fi-vi). In contrast, no prostatic buds appeared in β-catKO UGS tissues (Fig 4Gi-ii). IHC analysis using the epithelial markers E-cadherin and NKX3.1 further confirmed the absence of prostatic epithelial buds in the UGS tissues isolated from β-cat KO samples (Fig 4Giii-vi). Quantification of prostatic buds showed a significant decrease in the bud numbers in β-catKO UGS tissues compared to those in Ctrl UGS tissues (Fig 4I). These results demonstrate that the deletion of β-catenin expression in AR-lineage cells at E13.5 impairs early prostate development and bud formation.

### Deletion of β-catenin in embryonic AR-expressing cells impairs pubertal glandular morphogenesis and growth in the prostate

**Fig 5:**
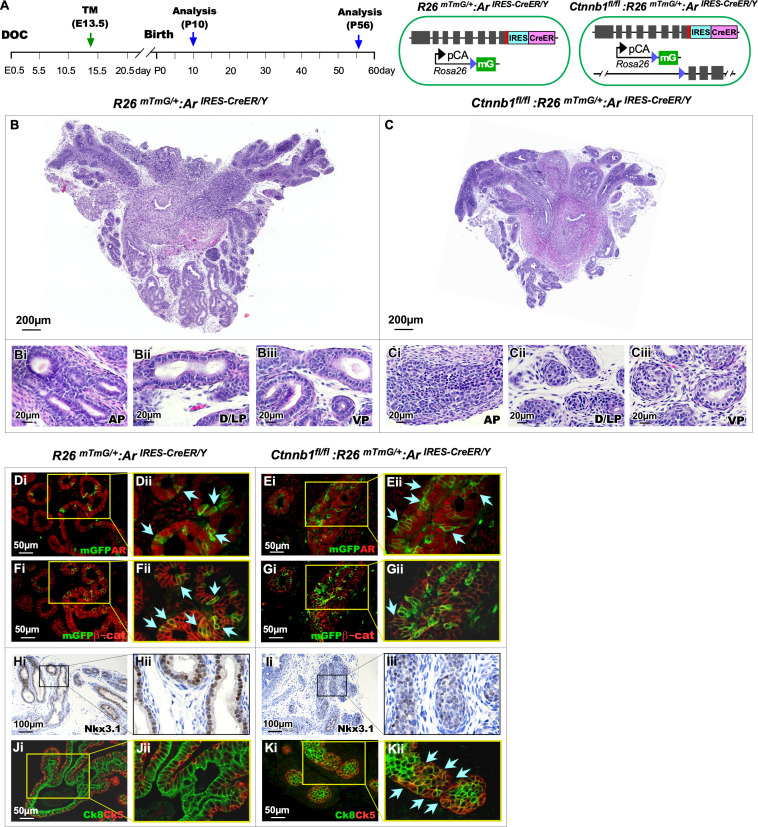
β-catenin is required in AR-lineage cells for prepubertal prostate development. (A) Experimental timeline and data collection timepoints, along with a schematic of the genetic construct showing Cre-mediated recombination following tamoxifen injection at E13.5. (B-C) H&E staining showing the histological features of the developing prostate in mice with β-catenin-competent and β-catenin-deficient AR-lineage cells. (Di-Gii) Immunofluorescence staining for mGFP, AR, and β-catenin demonstrating specific and efficient deletion of β-catenin in mGFP + , AR-lineage cells. (Hi-Iii) Immunohistochemical and (Ji-Kii) Immunofluorescence staining for prostate lineage-specific markers NKX3.1, CK8, and CK5, highlighting impaired epithelial differentiation resulting from β-catenin deletion in AR-lineage cells.

To evaluate the biological consequence of specific β-catenin deletion in embryonic AR-expressing cells during prostate formation, we administered Tamoxifen to pregnant female mice at E13.5, fostered both β-catenin-deficient and control newborns using wild-type females, and analyzed both β-catKO and control mice at P10 and P56 (Fig 5A). Histological analysis of prostate tissues isolated at P10 showed impaired prostate development in β-catKO mice, as evidenced by smaller prostate lobes than the controls (Fig 5B and 5C). While normal prostatic lobes appeared in prostate tissue sections of control mice (Fig 5Bi-iii), underdeveloped prostatic glands that contain small sizes of luminal cells and lack lumens within the glands were revealed in β-catKO prostate tissue sections, specifically in AP and DLP regions (Fig 5Ci-iii). Using Co-IF assays, we assessed the expression of AR and β-catenin in prepubescent AR-lineage cells in these prostate tissues. AR + mGFP+ cells were observed in both β-catKO and control prostatic tissue sections (light blue arrows, Fig 5Di-ii and 5Ei-ii). However, a significant reduction in mGFP + β-catenin+ cells was noted in prostate tissue sections of β-catKO mice compared to those of the control samples (Light blue arrows, Fig 5Fi-ii versus Fig 5Gi-ii), demonstrating the loss of β-catenin expression in AR-lineage cells. To further evaluate the effect of β-catenin deletion on prostatic epithelial cell differentiation, we assessed the expression of NKX3.1, a marker of prostatic budding cells and AR downstream target [25], in both β-catKO and control samples. In control prostate tissues, NKX3.1 exhibited uniform nuclear staining in prostatic luminal cells (Fig 5Hi-ii). In contrast, β-catKO prostate tissue sections showed fewer and irregular luminal epithelial cells within undeveloped prostate glands (Fig 5Ii-ii). Co-IF analysis showed that in control tissues, well-differentiated luminal epithelial cells stained for CK8 or basal epithelial cells stained for CK5 were clearly distinguishable in prostate glands (Fig 5Ji-ii). In β-catKO prostate tissues, however, many prostatic glandular cells co-expressed both CK5 and CK8, suggesting their intermediate cell properties. These results further demonstrate the critical role of β-catenin in AR-lineage cells for regulating prostatic postnatal epithelial differentiation and morphogenesis.

**Fig 6:**
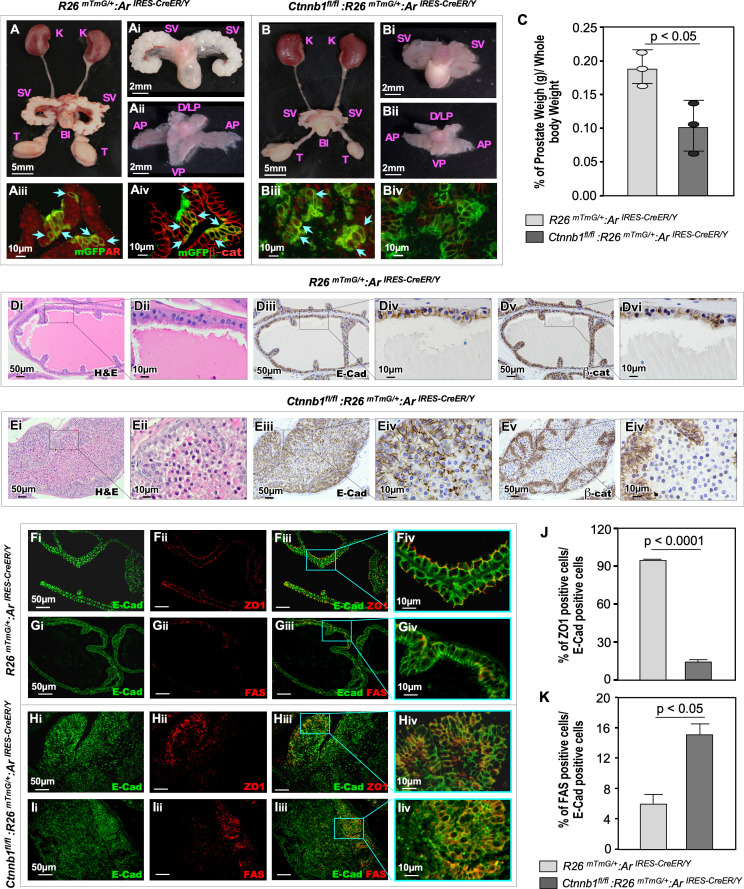
Loss of β-catenin in AR-lineage cells impairs postpubertal prostate morphogenesis and growth. (A-B) Representative images of urogenital tracts isolated from R26^*mTmG/+*^:Ar^CreER/Y^ and *Ctnnb1*^*fl/fl*^*:R26mTmG*^*/+*^*:Ar*^*CreER/Y*^. (C) Quantification of the prostate-to-body weight ratio, showing a significant reduction in prostate size following β-catenin loss in AR-lineage cells. (Di-Evi) H&E staining and immunohistochemistry of prostatic tissues from Ctrl and β-CatKO mice, revealing histopathological differences and differential expression patterns of epithelial markers. (Fi-Iiv) Immunofluorescence staining of the adherens junction protein ZO-1 and death receptor Fas, with their respective quantification shown in panels (J) and (K). Abbreviations: K: kidney; SV: seminal vesicle; Bl: bladder; T: testicle; AP: anterior prostate; D/LP: dorsal/lateral prostate; VP: ventral prostate.

To assess the effect of β-catenin loss in AR-lineage cells in response to pubertal androgen rise, we analyzed the prostate tissues isolated at P56 from both β-catKO and control mice (Fig 5A). Grossly, while the kidneys and other urogenital organs appeared normal in both male β-catKO and control mice (Fig 6A and 6B), β-catKO mice exhibited smaller seminal vesicles (SV) and prostates compared to their control counterparts (Fig 6Bi-ii versus Fig 6Ai-ii). Prostate weights in the β-catKO mice were significantly reduced relative to the controls (Fig 6C). Co-IF analysis showed co-expression of mGFP and AR proteins in prostate tissues isolated from both control andβ-catKO mice (light blue arrows, Fig 6Aiii and 6Biii). In contrast, while cell membrane staining for mGFP and β-catenin was evident in prostatic glandular cells of the control samples (light blue arrows, Fig 6Aiv), such staining was absent in those of β-catKO samples (Fig 6Biv). Histological analysis of the control prostate tissues showed normal prostate lobes and glandular elements, with uniform cell membrane staining for both E-cadherin and β-catenin in typical prostatic epithelial cells (Fig 6Di-vi). In contrast, the prostatic tissue samples isolated from age-matched β-catKO mice displayed abnormal glandular structures and lumen formation, with many cells filling the lumen, specifically within the AP regions (Fig 6Ei-ii). IHC analyses further revealed reduced E-cadherin staining and loss of β-catenin staining in the cells located in the mid-lumen region (Fig 6Eiii-vi). β-catenin plays a pivotal role in regulating cell adhesion as a component of adherens junctions (AJ) [26,27], and its deletion disrupts epithelial morphogenesis and results in loss of cell polarity [28,29]. To assess the role of β-catenin in maintaining prostatic cell polarity, we examined the expression of ZO1, a key component of AJ complex, in both control and β-catKO prostate tissues. In control samples, uniform cell membrane staining of E-cadherin overlaid with ZO1 staining appeared in each prostatic luminal epithelial cell exhibiting proper apico-basal cellular polarity (Fig 6Fi-iv). In contrast, prostate tissues from β-catKO mice showed reduced ZO1 staining overlapping with E-cadherin-positive cells (Fig 6Hi-iii), and abnormal ZO1 staining also appeared, indicating a loss of apico-basal cellular polarity in prostatic glandular cells (Fig 6Hiv). Quantitative analysis revealed a significant reduction in the proportion of ZO1-expressing cells among total E-cadherin-positive cells in β-catKO samples compared to the controls (Fig 6J). These findings demonstrate the critical role of β-catenin in regulating prostatic epithelial cell polarity, implicating an underlying mechanism for the abnormal lumen formation in β-catKO prostatic tissues (Fig 6Ei-ii). It has been shown that proper cell polarity and adherens junction formation prevent proapoptotic signals emanating from the Fas death receptor [30]. Therefore, we investigated whether the loss of apico-basal cellular polarity in β-catenin-deficient prostatic epithelial cells induces Fas signaling-induced cell deaths. Co-IF analysis showed a significant increase in Fas-expressing cells co-localizing with E-cadherin staining in β-catKO prostate tissues compared to the control mouse prostate tissues (Fig 6Ii-iv, 6Gi-iv). Furthermore, analysis of the proportion of prostatic Fas+ cells among total E-cadherin+ cells also showed a significant increase in β-catKO prostate tissue samples relative to the control samples (Fig 6K). Taken together, these experimental findings provide new insights into the regulatory role of β-catenin in controlling the pubertal morphogenesis and growth of prostatic AR-lineage cells.

## Discussion

The molecular mechanisms underlying androgen-induced interactions with other signaling pathways during early prostate development, postnatal morphogenesis, pubertal growth, and regeneration remain elusive. Additionally, the cellular properties and functions of prostatic AR-expressing cells that convey androgen signaling and act as prostatic progenitor cells in prostate differentiation and growth are largely unknown. One challenge contributing to these uncertainties is the lack of biologically relevant *in vivo* models to assess AR action during these biological events. Using gene-targeting approaches, we genetically modified the mouse *Ar* gene locus on the X chromosome to generate the *Ar* ^*IRES-CreER*^ allele, in which both AR and CreER proteins are co-expressed within the same cells. By combining this allele with the mTmG reporter mice [17], this new mouse model allows us to genetically label AR-expressing cells in a spatial and temporal manner, and trace the fate of these AR-expressing cells and their descendants throughout prostate development, morphogenesis, and gland formation.

As detailed in this study, the embryonic AR-expressing cells labeled at E13.5 were detected in both UGM and UGE regions at E18.5, consistent with the previous studies. Moreover, the mGFP-expressing cells were further detected at postnatal days 10 and 56, as well as after three cycles of prostatic regeneration induced by repeated androgen deprivation and replacement (Fig 2). These results directly demonstrate that the AR-expressing cells labeled at E13.5 transmit the mGFP expression to their descendant cells during embryonic, prepubescent, and pubertal stages. Additionally, we examined the cellular properties and regenerative potential of AR-lineage cells derived from embryonic AR-expressing cells sustained over multiple castration-regeneration cycles (Fig 3). Co-IF analyses further demonstrated the high proliferative properties of these mGFP-expressing cells, implicating the prostatic progenitor properties of AR-lineage cells derived from embryonic AR-expressing cells. Collectively, these findings suggest that the *Ar* ^*IRES-CreER*^ allele is a valuable tool for labeling AR-expressing cells at the specific developmental stages to trace the fate and expansion of their descendants throughout early prostate development, prepubescent morphogenesis, pubertal growth, and regeneration.

Canonical Wnt signaling pathways have been shown to play a significant role in early prostate development, morphogenesis, and growth [21–23]. Deletion of either β-catenin or AR expression alone in prostatic Wnt-responsive Axin-expressing cells impairs early prostate development and gland formation [23,24]. Because the expression of the *CreER* recombinase is driven by the endogenous *A*r promoter and can be activated by Tamoxifen administration in the *Ar* ^*IRES-CreER*^ allele, it can be employed to perturb and manipulate androgen action-mediated effects on other signaling pathways through the incorporation of additional floxed alleles. Therefore, we directly addressed a significant but unknown question regarding the regulatory role of β-catenin in AR-expressing cells in controlling prostate cell fate and growth using this *Ar* ^*IRES-CreER*^ allele in this study. Analysis of UGS tissues from β-catKO embryos isolated at E18.5, administrated with Tamoxifen at E13.5, showed impaired UGE early development and bud formation, which provides new experimental evidence demonstrating the regulatory role of Wnt/β-catenin in AR-expressing cells during early prostate epithelial development. Further examination of prostate tissues isolated from β-catKO mice at prepubescent and post pubertal stages also identified additional impairments in prostate glandular development, morphogenesis, and growth. Specifically, β-catenin-deficient AR-lineage cells within the prostate glands of β-catKO mouse samples appeared irregular and lacked typical luminal epithelial morphology. Abnormal expression of ZO1, a component of the AJ that directly contributes to the apical-basal cell polarity, was identified in prostate tissue samples of β-catKO mice, resulting in a loss of the cell polarity in these β-catenin-deficient AR-lineage cells. Our findings are consistent with the previous reports showing that altered β-catenin expression can impair stem and progenitor cell morphogenesis and result in loss of cell polarity as observed in different tissues and organs of many species [29,31,32].

It has been shown that maintaining normal AJ formation and cell polarity is essential for preventing proapoptotic signals emanating from the Fas death receptor [30]. Specifically, the cell adhesion complexes composed of E-cadherin, β-catenin, actin, and other components can sequester Fas-mediated cell signaling. Using co-IF approaches, we directly assessed the expression of Fas in unpolarized prostate epithelial cells derived from β-catenin-deficient AR-lineage cells. A significant increase in Fas-expressing epithelial cells was observed in prostate tissue sections of β-catKO mice in comparison to those from control mice. These observations, in combination with other results presented in this study, implicate a regulatory mechanism underlying loss of β-catenin-induced impairment in prostate epithelial development, morphogenesis, and growth.

In this study, we reported the newly developed *Ar* ^*IRES-CreER*^ allele and its validation in combination with the mTmG reporter and the floxed allele of the *Ctnbb1* gene, encoding β-catenin. The data presented in this study demonstrate that Tamoxifen administration can sufficiently and specifically induce the activation of the CreER recombinase drive by the endogenous *Ar* promoter in a temporal and spatial manner. The characteristics of this mouse model demonstrate the relevance and significance of this new genetic tool that allows us to visualize and assess AR-mediated signaling pathways throughout the development, morphogenesis, and growth of the prostate, as well as other tissues and organs.

## Materials and methods

### Ethics statement

All animal procedures were approved by the Albert Einstein College of Medicine Animal Care and Use Committee (IACUC).

**Mouse generation, mating, and genotyping.** *Rosa26*^*mTmG/*+^ *(R26*^*mTmG/*+^) reporter mice were kindly provided by Dr. Liqun Luo [17]. The *Ctnnb1*^*fl/fl*^ mice were obtained from the Jackson Laboratories (stock #4152) [33].

To generate the *Ar*^*IRES-CreER*^ mice, targeting vectors were designed based on the previous report for making *Ar*^*IRES-PLAP-IRES-nLacZ*^ *mice* [34]. The IRES-CreER fragment was generated by PCR from the IRES-mCherry plasmid (Addgene #80139) and assembled accordingly. This fragment was inserted into the region after the stop codon of the mouse *Ar* gene. Three guide RNAs (gRNA) were designed for this insertion site (AAGTGCCCAAGATCCTTTCT, TTCCACACACAGTGAAGATT, and TTCCAAATCTTCACTGTGTG) and synthesized via *in vitro* transcription. CAS9 protein was purchased from PNA Bio [8]. The IRES-CreER donor DNA (dDNA) fragment was cloned into the targeting vector and flanked by left and right homologous arms (approximately 1kb each), which were generated by PCR using C57BL/6J mouse genomic DNA as template with the following primer pairs: AR-5F: TTCCAGTGGATGGGCTGAAAAATC, AR-5R: ACGCGTTCTTCACTGTGTGTGGAAATAGATGGGCTTGACTTTGCCAGAAAGGATCTTGGGCACTTG, AR-3F: TTTGGAAACCCTAATACCC, and AR-3R: CAAAGAGTCAGACCTTTCC. A point mutation was introduced by the AR-5R primer at the gRNA PAM site to prevent cleavage of the donor DNA. C57BL/6J mice (4–6 weeks old) were used for zygote collection. The guide RNAs, CAS9 protein, and donor DNA were mixed at a ratio of 5 ng/uL: 15 ng/uL: 3 ng/uL before injection into mouse zygotes. Injected zygotes were implanted into CD1 foster mothers. Genomic DNA from offspring tail tissues was isolated and analyzed by genomic DNA PCR using the following primers AR-fw1: AAGTCCTTCATTCTGAATTCCCCAGACA; CreER-Rev1: ATCCCTGAACATGTCCATCAGGTTCTT; CreER-fw6: GTCCAATTTACTGACCGTACACCAAAATTTGCC; and AR-3rev1: GAGGAAAGGATGATTGCACTATGGCATC. The PCR DNA sequences were verified by Sanger sequencing. Confirmed *Ar* ^*IRES-CreER*^ founder mice were then backcrossed with wild-type C57BL/6J for two to three generations to eliminate mosaicism resulting from CRISPR/CAS9-based gene editing. Experimental mice were generated by intercrossing *Ar*^*IRES-CreER/X*^ female mice with either *R26*^*mTmG/*+^ male mice or *Ctnnb1*^*fl/f*^;*R26*^*mTmG/*+^ male mice.

To induce genetic recombination at embryonic day 13.5 (E13.5), pregnant female mice received an intraperitoneal injection of tamoxifen (125 µg/g body weight; Sigma) suspended in corn oil (Sigma) as previously described [23]. Genotyping for the *Ar*^*CreER*^ allele was performed using the following primers: forward 1, (F1) AGTCCC ATA TGG TGA GCG TG, forward 2, (F2) TAC ATG CGC CCA CTA GCC and (reverse, R) AGG AAC CTG TTC ACG ACA GAC. Genotyping of the *Ctnnb1* and *mTmG* alleles was conducted as previously reported [33].

For prostate regression and regeneration assays, adult male mice were castrated at postnatal days 56 (P56), as described previously [20], and maintained in a castrated state for 4 weeks. This was followed by 4 weeks of testosterone supplementation (round 1). The similar cycle of castration and androgen supplementation was repeated for two additional rounds. Testosterone supplementation was achieved by subcutaneous implantation of a 25 mg testosterone pellet (Innovative Research of America) in the dorsal region of the mice. To label proliferating cells during prostate development, 5-bromo-2’-deoxyuridine (BrdU) (80 mg/kg; Sigma) was administered via intraperitoneal injection either once or once daily over three consecutive days, depending on the experimental requirement.

**Histology, immunostaining, and mT/mG assays.** Mouse tissues were fixed in 10% neutral-buffered formalin (American Master Tech Scientific), then processed and embedded in paraffin, or processed for OCT embedding following overnight fixation in 10% neutral-buffered formalin and cryoprotection in 1X PBS (pH 7.3) containing 30% sucrose at 4°C, as previously described [23]. Paraffin- or OCT-embedded tissue blocks were cut into serial sections of 4 μm and 5 μm respectively and used for hematoxylin-eosin (H&E) staining for subsequent histological characterization [23]. Immunohistochemistry (IHC) was performed as described previously [23]. In brief, IHC was performed following deparaffinization when required and rehydration of tissue sections through a decreasing ethanol gradient (100% to 50%). Heat-mediated epitope retrieval was performed in 0.01M citrate buffer (pH 6.0), followed by quenching endogenous peroxidase activity for 15 min in 0.3% H_2_O_2_ in methanol. Tissues sections were then washed and incubated at room temperature for 1h in 1X PBS (pH 7.3) containing 5% goat or donkey serum to block nonspecific binding, followed by overnight incubation at 4°C with primary antibodies diluted in 1% goat or donkey serum in 1X PBS. The following day, sections were washed with 1X PBS and incubated for 30 minutes with streptavidin-conjugated horseradish peroxidase (Strep-HRP; SA-5004, Vector Laboratories; 1:500 dilution) and developed using a 3,3’-diaminobenzidine (DAB) kit (SK-4100, Vector Laboratories). Slides were counterstained with 5% Harris Hematoxylin, dehydrated through an ascending ethanol gradient, and mounted with Permount Medium (SP15–500, Fisher Scientific).

For immunofluorescence (IF) staining, the procedures described above were followed with the exception that the incubation in H_2_O_2_ in methanol was omitted. Tissues sections were incubated with fluorescently conjugated secondary antibodies targeting the primary antibodies of interest, washed, and then mounted using Vectashield Mounting Medium with DAPI (H-1200, Vector Laboratories). Membrane-bound Tomato (mT) and membrane-bound green fluorescent protein (mGFP) signal detection on OCT sections was performed as previously described [23]. In brief, tissue sections from OCT-embedded samples were rinsed with PBS (pH 7.3) and mounted with Vectashield Mounting Medium with DAPI (H-1200, Vector Laboratories) for imaging. A complete list of antibodies used for both IHC and IF is provided below: anti-mGFP (1:500, Rabbit mAb CST #295; 1:500, Mouse mAb CST #2955), anti-E-Cad (1:500, Rabbit mAb CST #3195; 1:200 Mouse mAb CST #14472), anti-AR (1:500, Invitrogen, Catalog #PA1–9005), Anti-α-SMA (1:200, Sigma, #A5228), anti-VIM (1:2000, BioLegend, #919101), anti-CK8 (1:2000, Mouse mAb, BioLegend #904804; 1:200, Rabbit pAbs, ab59400), anti-CK5 (1:3000, Rabbit pAbs, BioLegend #905503), anti-NKX3.1 (1:500, Athena Enzyme Systems™, #0315), anti-β-catenin (1:200, BD Transduction Laboratories #610154), anti-ZO1 (1:500, Rabbit pAbs, Proteintech #21773–1-AP), anti-Fas (1:30, Goat pAbs, Bio-Techne #AF435), anti-BrdU (1:200, Mouse mAb CST #5292), and anti-CD45(1:500, Rabbit pAbs, #ab10558).

**Microscope image acquisition.** H&E and IHC images were acquired using a Zeiss Axio Lab A1 microscope equipped with 5x, 10x, 20x, and 40x A-Plan objectives. A Canon EOS 1000D camera and Axiovision software (Carl Zeiss, Oberkochen, Germany) were used for images capture. IF, BrdU, and mTmG signals were acquired on an Olympus IX81 Epi-Fluorescence Microscope using 10x, 20x, and 40x Nikon Plan Fluor objectives. These images were recorded with a QImaging RETIGA EXi camera and processed using QCapture (QImaging) and Zen software (v3.9; Carl Zeiss Microscopy GmbH).

**Statistical analysis.** Graphs were generated using GraphPad Prism version 9 (GraphPad Software, LLC), and data are presented as mean ± standard deviation (SD). Statistical analyses were performed using unpaired t-tests, multiple t-tests, or one-way ANOVA followed by Tukey’s post hoc test for multiple group comparisons. A p-value or a false discovery rate (FDR) of less than 0.05 was considered indicative of statistical significance.

## Supporting information

S1 File Raw numerical values and summary statistics for graphs shown in Figs 1M, 2J, 3K, 4H, 4I, 6C, 6J and 6K. (XLSX)
